# Determination of the relative economic impact of different molecular-based laboratory algorithms for respiratory viral pathogen detection, including Pandemic (H1N1), using a secure web based platform

**DOI:** 10.1186/1743-422X-8-277

**Published:** 2011-06-06

**Authors:** Bonita E Lee, Shamir N Mukhi, Jennifer May-Hadford, Sabrina Plitt, Marie Louie, Steven J Drews

**Affiliations:** 1 University of Alberta, Edmonton AB, Canada; 2 Canadian Network for Public Health Intelligence, Public Health Agency of Canada, Winnipeg, MB, Canada; 3 Public Health Agency of Canada, Ottawa, Ontario, Canada; 4 University of Calgary, Calgary, AB, Canada; 5 Provincial Laboratory for Public Health, Alberta, Canada

**Keywords:** influenza, testing, relative comparisons, test algorithms, economic impact

## Abstract

**Background:**

During period of crisis, laboratory planners may be faced with a need to make operational and clinical decisions in the face of limited information. To avoid this dilemma, our laboratory utilizes a secure web based platform, Data Integration for Alberta Laboratories (DIAL) to make near real-time decisions.

This manuscript utilizes the data collected by DIAL as well as laboratory test cost modeling to identify the relative economic impact of four proposed scenarios of testing for Pandemic H1N1 (2009) and other respiratory viral pathogens.

**Methods:**

Historical data was collected from the two waves of the pandemic using DIAL. Four proposed molecular testing scenarios were generated: A) Luminex respiratory virus panel (RVP) first with/without US centers for Disease Control Influenza A Matrix gene assay (CDC-M), B) CDC-M first with/without RVP, C) RVP only, and D) CDC-M only. Relative cost estimates of different testing algorithm were generated from a review of historical costs in the lab and were based on 2009 Canadian dollars.

**Results:**

Scenarios A and B had similar costs when the rate of influenza A was low (< 10%) with higher relative cost in Scenario A with increasing incidence. Scenario A provided more information about mixed respiratory virus infection as compared with Scenario B.

**Conclusions:**

No one approach is applicable to all conditions. Testing costs will vary depending on the test volume, prevalence of influenza A strains, as well as other circulating viruses and a more costly algorithm involving a combination of different tests may be chosen to ensure that tests results are returned to the clinician in a quicker manner. Costing should not be the only consideration for determination of laboratory algorithms.

## Background

The influenza pandemic of 2009-2010 was probably the most-prepared for pandemic in Canadian history [1]. In Canada, much of this preparedness relied on the use of molecular technologies for the detection of viral pathogens, including influenza. These tests are highly sensitive for the detection of viral pathogens when compared to traditional culture based or antigen-detection methods [2-4]. A key dilemma of commercial multiplexed versions of these tests, which detect multiple pathogens, is the relatively high cost. In contrast, "home-brew" methods to detect influenza are often less costly but cannot detect other respiratory viral pathogens thus may have limited applications when other respiratory viral pathogens are circulating and/or the prevalence of influenza is low. Moreover, at the start of the pandemic, the performance characteristics of various diagnostic assays remained unknown [5]. Thus, effective laboratory planning for a pandemic response should take into account factors such as test characteristics, test cost, prevalence of pathogens and the diagnostic and clinical requirements of the end user.

The Province of Alberta, Canada has a population of 3.7 million (est. July 2010) [6] and the Provincial Laboratory for Public Health, ProvLab, supports the pandemic plans of this jurisdiction and surrounding Northern Territories by providing molecular detection of Influenza A as well as other respiratory viral pathogens. Real-time and retrospective data analysis and integration of laboratory results within ProvLab is enabled through a secure, interactive web based platform called DIAL (Data Integration for Alberta Laboratories), which enables customized trending (graphs, tables, or map) including integrated multi-virus analysis and quick data extraction of cleaned and interpreted respiratory virus data generated at ProvLab [7].

ProvLab has access to monoplex assays designed only for influenza A and B as well as multiplexed assays for influenza A and B and other respiratory viral pathogens. Influenza subtype analysis for seasonal H1 and H3 influenza is available on monoplex assays and on multiplexed respiratory viral pathogen panels [8]. This laboratory also utilizes "home-brew" monoplex assays for the confirmation of the Pandemic (H1N1) 2009 virus [9].

During a pandemic, one of the key issues facing diagnostic testing laboratories is how to utilize laboratory tests in the most cost-effective manner given the incredible workloads. This manuscript will utilize the data collected by the DIAL system as well as laboratory test cost modeling to identify the most cost-effective of four proposed scenarios of testing for Pandemic H1N1 (2009) and other respiratory viral pathogens. Beyond cost, advantages and disadvantages to each scenario will be outlined.

## Methods

### Collection of historical data from two waves of the pandemic

ProvLab was responsible for the majority of respiratory virus detection within the Province. Raw laboratory testing data for respiratory virus detection interpreted as clinically meaningful respiratory virus targets were accessible real-time on DIAL. Data for the prevalence of respiratory viral pathogens was calculated on a weekly basis from April 19, 2009 to April 24, 2010.

### Molecular tests included in the analysis

Prior to the pandemic, the Luminex respiratory virus panel (RVP) was used at ProvLab to detect respiratory virus on all DFA negative nasopharyngeal and lower respiratory virus samples [9]. Real-time RT-PCR for influenza A M gene (CDC-M), real-time RT-PCR for influenza A seasonal subtypes H1 and H3, and ProvLab developed real-time RT-PCR for Pandemic H1N1 (2009), were rapidly implemented in the beginning of April 2009 as part of the Pandemic preparedness plan [5]. Performance characteristics, *sensitivity *and *specificity*, of the different assays for Pandemic H1N1 (2009) were determined by analyzing the data extracted from DIAL. The estimated sensitivities of each method for influenza A virus were 98.6% (CI 96.5-99.6%) for CDC-M and 77.3% (CI 72.1-82.1%) for RVP if equivocal influenza A result by RVP were considered as negative for influenza A with no further testing [10]. The specificity was 99.8% (CI 99.7-99.9%) for CDC-M and 99.8% (CI 99.8-99.9%) for RVP.

### Molecular testing algorithms

Four approaches were included to estimate costs in the laboratory using historical data. Scenario A was the testing algorithm used from April 19, 2009 till June 23, 2009 whereby Scenario B was adopted after the analysis of the data showed a higher sensitivity of CDC-M for influenza A as compared to RVP.

**Figure 1:**
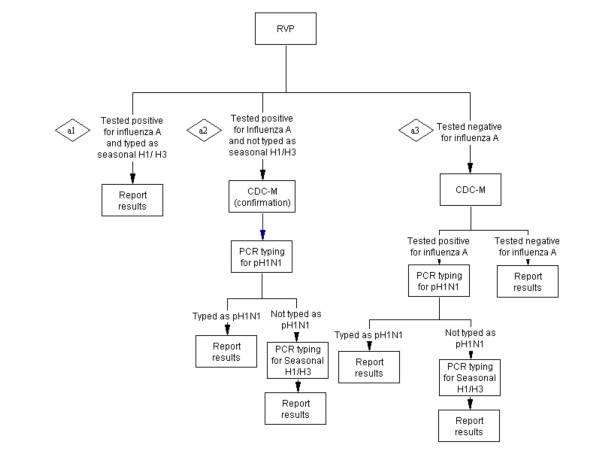
**Scenario A - RVP with/without CDC-M**.

**Scenario A - RVP first with/without CDC-M** (Figure 1): Testing for respiratory viruses by RVP first, and if typed as seasonal influenza A then stop. If influenza A-positive specimen is not-typeable on RVP, then perform CDC-M to confirm influenza result as well as typing using real-time RT-PCR by first screening for pH1N1, followed by subtyping for seasonal H1/H3 if negative for pH1N1. If specimen is influenza A-negative then attempt influenza A detection by CDC-M protocol with sub-typing of influenza A positive samples

**Figure 2:**
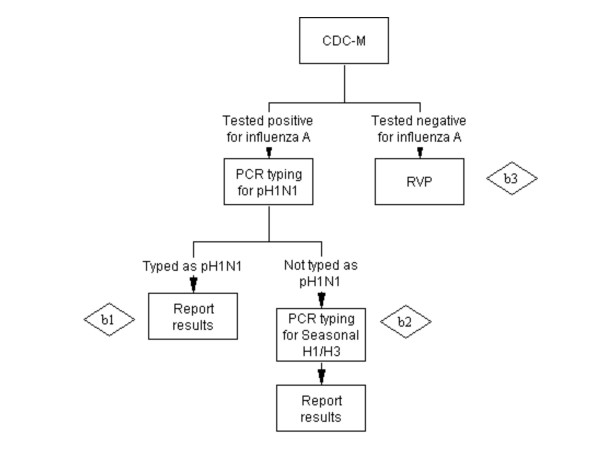
**Scenario B - CDC-M with/without RVP**.

**Scenario B - CDC-M first with/without RVP** (Figure 2): Testing for influenza A by CDC-M first with subtyping of influenza A positive samples and the testing of influenza A-negative specimens by RVP.

**Figure 3:**
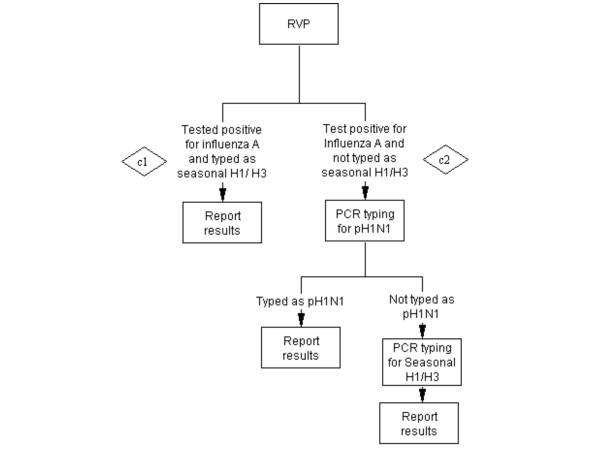
**Scenario C - RVP only**.

**Scenario C - RVP only** (Figure 3): Only testing for influenza and respiratory viruses by RVP. If influenza A positive and typed as seasonal influenza by RVP, then stop. If influenza A-positive specimen is not-typeable on RVP, perform typing pH1N1 +/- seasonal H1/H3. If influenza A is negative by RVP then no more testing.

**Figure 4:**
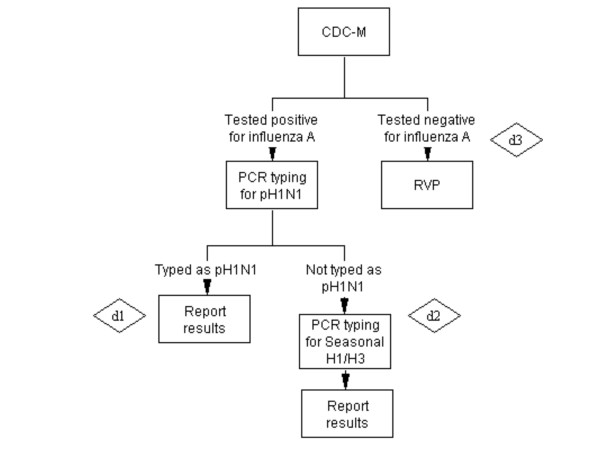
**Scenario D - CDC-M only**.

**Scenario D - CDC-M only** (Figure 4): testing only for influenza A by CDC-M, followed by pH1N1 typing and then seasonal influenza typing in pH1N1-negative.

### Estimation of costs for various test algorithms

Costs estimates of different testing algorithms (Figures 1,2,3,4) were generated from a review of historical costs in the laboratory and were based on 2009 Canadian dollars. Factors include; total number of respiratory virus specimens; expected % specimen positive for influenza A detected by RVP; expected % specimen positive for pH1N1, and expected % positive for seasonal H1 and H3 taking into account a proportion of specimens that tested positive for influenza A and not typeable because of low viral titers; and % influenza A positive tested negative by RVP but detected by CDC-M. Key assumptions are; 1) costs include labor and reagent and not overhead costs, 2) costs do not include shipment, billing costs, nor data entry 3) costs include extraction plus scenario of test indicated, 4) costs are for molecular testing only and do not include culture nor DFA, and 5) the number of false-positive and false-negative test results will be negligible and were not included in the cost analysis. The weekly total cost were calculated using actual number of specimens tested at ProvLab and the numbers of influenza positive specimens and their H-types during the study period. For the analysis, weekly relative cost of the different algorithms was calculated at each point in time for the study period as a ratio of cost for each algorithm to the cost of the algorithm with the lowest cost for each respective week over the study period.

Formulas for Cost estimations:

#### A. Scenario A - RVP with/without CDC-M: Cost of a1 + Cost of a2 + Cost of a3 (figure 1)

Cost of a1: Cost of RVP*No. of specimen*Proportion of influenza A positive samples detected by RVP*Proportion of influenza A positive samples that are seasonal H1/H3

Cost of a2: (Cost of RVP*No. of specimen*Proportion of influenza A positive samples detected by RVP)*(*1-Proportion of influenza A positive samples that seasonal H1/H3*) + [(*Cost of CDC-M *+ Cost of pH1N1 typing)*No. of specimen*Proportion of influenza A positive samples detected by RVP*Proportion of influenza A positive samples that are pH1N1)] + [(*Cost of CDC-M *+ Cost of pH1N1 typing+ Cost of seasonal H1N1 typing)*No. of specimen*Proportion of influenza A positive samples detected by RVP*(*1-Proportion of influenza A positive samples that are pH1N1 and seasonal H1/H3*)]

Cost of a3: [(Cost of RVP+ Cost of CDC-M)*No. of specimen*Proportion of influenza A positive samples tested negative by RVP and positive by CDC-M] + [Cost of pH1N1 typing*No. of specimen*Proportion of influenza A positive samples tested negative by RVP and positive by CDC-M *Proportion of influenza A positive samples that are pH1N1] + [(Cost of pH1N1 typing+ Cost of seasonal H1N1 typing)*No. of specimen*(*1-Proportion of influenza A positive samples that are pH1N1 and seasonal H1/H3*)]

#### B) Scenario B - CDC-M with/without RVP: Cost of b1 + Cost of b2 + Cost of b3 (figure 2)

Cost of b1: (Cost of CDC-M+ Cost of pH1N1 typing)*No. of specimen*Proportion of influenza A positive samples detected by CDC-M*Proportion of influenza A positive samples that are pH1N1

Cost of b2: (Cost of CDC-M+ Cost of pH1N1 typing+ Cost of seasonal H1/H3 typing)*No. of specimen*Proportion of influenza A positive samples detected by CDC-M*Proportion of influenza A positive samples that are SeasonalH1/H3

Cost of b3: (Cost of RVP+ Cost of CDC-M)*No. of specimen*(1- Proportion of influenza A positive samples detected by CDC-M)

#### C) Scenario C - RVP only: Cost of c1 + Cost of c2 (figure 3)

Cost of c1: Cost of RVP*No. of specimen*Proportion of influenza A positive samples detected by RVP*Proportion of influenza A positive samples that are seasonal H1/H3

Cost of c2: (Cost of RVP*No. of specimen*Proportion of influenza A positive samples detected by RVP)*(*1-Proportion of influenza A positive samples that seasonal H1/H3*) + (Cost of pH1N1 typing*No. of specimen*Proportion of influenza A positive samples detected by RVP*Proportion of influenza A positive samples that are pH1N1) + [(Cost of pH1N1 typing+ Cost of seasonal H1N1 typing)*No. of specimen*Proportion of influenza A positive samples detected by RVP*(*1-Proportion of influenza A positive samples that are pH1N1 and seasonal H1/H3*)]

#### D) Scenario D - CDC-M only: Cost of d1 + Cost of d2 + Cost of d3 (figure 4)

Cost of d1: (Cost of CDC-M+ Cost of pH1N1 typing)*No. of specimen*Proportion of influenza A positive samples detected by CDC-M*Proportion of influenza A positive samples that are pH1N1

Cost of d2: (Cost of CDC-M+ Cost of pH1N1 typing+ Cost of seasonal H1/H3 typing)*No. of specimen*Proportion of influenza A positive samples detected by CDC-M*Proportion of influenza A positive samples that are SeasonalH1/H3

Cost of d3: (Cost of CDC-M)*No. of specimen*(1-Proportion of influenza A positive samples detected by CDC-M)

## Results

**Figure 5:**
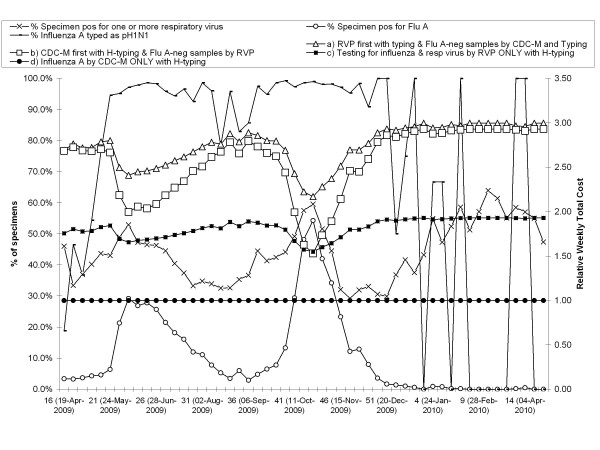
**Rate of specimens positive for respiratory virus(es), influenza A and Pandemic H1N1 (2009) and the relative total weekly test cost per specimen for Scenarios A-D during April 19 2009 to April 24, 2010**. The relative cost of each algorithm is calculated as the ratio of each algorithm to the lowest cost of any algorithm for the same time period.

The median weekly number of specimens tested at ProvLab during the study was 682 (range: 293-3,876). The median % of specimens tested positive for one or more respiratory virus during the study using Scenario A till June 23, 2009 and Scenario B for the remaining period was 44.2% (range: 29.2-63.9%). Two peak periods (mid-May to mid-August) and (late-September to early-December) were observed for pH1N1 when the weekly positive rates for influenza A were 29.2% and 53.4% respectively. Figure 5 shows the relative weekly total costs of each proposed scenario given the prevalence of pH1N1 in our jurisdiction during both pandemic waves of 2009 and the pandemic interwave period.

From Figure 5 it is evident that Scenario D (influenza A RT-PCR and subtyping alone) was the least expensive method throughout both waves of the pandemic and the inter-wave period and the relative weekly total cost of this scenario equaled to one. In contrast, the next least expensive approach was Scenario C (Testing by RVP followed by influenza pH1N1 sub-typing). Both Scenario A (RVP first, followed by RT-PCR for influenza A on negative specimens) and Scenario B (influenza A RT-PCR followed by RVP on influenza A-negative specimens) had similar costs when the rate of influenza A was low (< 10%) and Scenario A had higher relative cost as the incidence of influenza peaked during the pandemic. Scenario A would have provided more information of specimens with influenza A with mixed respiratory virus infection as compared with Scenario B. On the other hand, the turn-around time (TAT) of RT-PCR for influenza A is 24 hours where as the TAT of RVP is 48 hours, thus Scenario B will provide a faster report for influenza A positive specimens. Further to timing issues, the hands-on labor impact of the RVP assay is greater than that of the RT-PCR for influenza.

## Discussion

This manuscript has modeled several scenarios for the detection of Pandemic (H1N1) 2009, with the two most expensive scenarios being Scenario A and Scenario B. Pros and cons of different scenarios are shown in Table 1. Scenario A (RVP with/without CDC-M) consistently had the highest costs that were maintained throughout the whole pandemic period, although costs did drop as the % of specimens positive for influenza A increased. Not only were the costs high for Scenario A, the labor intensive nature of the assay and increased turn-around time for influenza A compared to other methods would make it difficult to deliver a timely answer to clinicians when compared to other scenarios. The costs for Scenario B (CDC-M first with H-typing and FluA-neg samples by RVP) was the second most expensive algorithm throughout the pandemic with costs dropping as the % of specimens positive for influenza A increased. However, the up-front use of the CDC-M assay would enable influenza diagnosis to be undertaken with less technologist hands-on time and a quicker turn-around time than RVP based methods. Test cost and increased technologist hands-on-time would occur during periods outside the influenza A peak, and diagnosis of other respiratory viruses would still be dependent on the use of the RVP.

**Table 1:** Benefits of using each influenza A testing scenario during the pandemic

Scenario	Description	Pros	Cons	Other comments
A	RVP first with typing &Flu A-neg* samples by CDC-M and Typing * Flu A positive samples not typed by RVP were also tested by CDC-M for confirmation	• Provide data on the performance characteristics of different diagnostic assays • Identifies mixed influenza and other respiratory infections • Surveillance for other respiratory viruses	• Argument about relevance of diagnosing other viruses apart from RSV A/B during a public health emergency • Resource intense • Labor intensive • May be difficult to stock for pandemic • Poor turn-around time for influenza A	• After obtaining data for validation purposes and total virus surveillance, this approach was replaced by a more cost-effective and time sensitive diagnostic approach (Scenario B) which does not provide the prevalence of mixed infection with influenza A

B	CDC-M first with H-typing & FluA-neg samples by RVP	• Quicker Turn-around-time to influenza diagnosis compared to RVP-based assays • Scenario drops out more labor intensive test as % influenza increases • Influenza-negative specimens still being tested for other respiratory viruses	• Will not identify influenza co-infections with other viruses • May still be considered as resources intense • Argument about relevance of diagnosing other viruses apart from influenza A during a public health emergency	• Some laboratories may find this approach too resource intense. • Relative cost decrease as % specimen positive for influenza increases

C	RVP only with H-typing	• Diagnosis of mixed infections	• Questionable use for influenza surveillance • Labor intensive • May be difficult to stock for pandemic • Poor turn-around time for influenza A	• Possible use when no influenza circulating or influenza prevalence <5% or acceptance of having missed influenza cases because of lower sensitivity of RVP

D	influenza A by CDC-M only with H-typing	• Less labor-intensive, • Lower cost than RVP • Quicker Turn-around-time compared to RVP-based assays • Excellent tool for testing during peak pandemic period	• Does not allow for identification of other circulating viruses or co-infections	• Depends on high prevalence of influenza A and lower prevalence of other viruses or mixed infections • Role when maximum peaks are seen (>60% specimen influenza A)

The two less costly algorithms were Scenario C and Scenario D. Scenario C (testing for influenza and respiratory virus by RVP only with H-typing) was the third lowest cost over all pandemic phases with costs crossing over with Scenario B at the peak. This algorithm would be focused on using the labor-intensive RVP assay and would require a longer turn-around-time to influenza A diagnosis than Scenario B. Furthermore, there have been some recent questions as to the use of RVP for the primary diagnosis of influenza A in patient specimens [10]. The least expensive scenario across all phases of the pandemic was Scenario D (Influenza A by CDC-M only with H-typing). This scenario was also less labor intensive than scenarios focused on RVP and provided a quicker turn-around time to the diagnosis of influenza A than scenarios using RVP as the primary diagnostic tool. However, this scenario does not provide any data on other circulating respiratory viruses which had both diagnostic and surveillance value.

Outright costs alone should not be the primary driving force during the decision process regarding which algorithm is used. Other relevant factors in this decision-making process include needs for test performance data at the start of the pandemic, test turn-around-time, burden on laboratory staffing hours, availability of commercial kits, space available to carry out assays, and overall algorithm performance characteristics [10]. Thus, laboratorians may decide to provide tests that are more costly to the laboratory but provide better patient care and may even save costs globally in patient care systems [11]. These increased laboratory test cost per specimen may be recovered in increased efficiency in other areas such as clinical decision making, infection control and public health practice [12,13].

The benefit of testing for other respiratory viruses needs to be determined by each laboratory following discussion with its client bases. These and other authors have often experienced that some laboratorians and clinicians believe that there is little benefit to testing viruses other than influenza due to the lack of widespread antivirals for other viral pathogens [14]. However, testing for other viruses fulfills key surveillance roles and may have patient care and economic benefits in some settings [15-17]. Steps such as cohorting patients based on viral etiology may be found by some institutions to ease the clinical management of patients [18]. Identification of other viruses my also play a role in the discontinuation of antimicrobial therapy and decreased antibiotic use in some clinical settings [12].

The identification of trends in viral was easy and readily accessible through the use of DIAL which is a partnership between Alberta's Provincial Laboratory for Public Health (ProvLab) and the Canadian Network for Public Health Intelligence (CNPHI). DIAL was founded by Drs. Jutta Preiksaitis, Bonita Lee and Shamir Mukhi in 2007 to address critical problems in extracting and managing laboratory-based data and was created so that ProvLab staff and other stakeholders would have an easier method for extracting, interpreting and analyzing laboratory data. Historically at ProvLab, the extraction of laboratory data from the Provlab information system (COHORT) was complex and could only be performed by computer programmers. Therefore, data was not easily accessible to laboratory staff, public health practitioners or other stakeholders. Moreover, the extracted data still required interpretation by laboratory experts to convert it into clinically meaningful final result. However, DIAL provides a solution to these problems by providing a simple web-based interface that enables users to access, summarize and analyze cleaned and interpreted real-time laboratory data [7].

Pandemic preparedness involves not only the technical preparation of laboratories but also an understanding of both the cost implications of test utilization as well as the characteristics of each test algorithm. This manuscript indicates that a single methodology is not applicable to all conditions and that test characteristics may be as or more important than test-cost. Also it is notable that the cost of tests per specimen will vary depending on the prevalence of influenza A as well as other circulating viruses. Thus as the prevalence of influenza increases, an RVP only strategy (Scenario C) will increase in cost while a strategy that primarily using the CDC protocol with or without RVP (Scenario B) will decrease in cost. Clinician as well as patient needs may also have an impact on which algorithm is chosen as some situations may require quick turn-around-times (e.g. detection of influenza) while others may require an more comprehensive assessment of other respiratory viruses (e.g. cohorting patients with common respiratory infections) [19-21]

This manuscript indicates that an ideal pandemic plan should allow for the laboratory to effectively shift between different algorithms as the pandemic progresses and depending on whether there is a need to identify other respiratory viral pathogens. The diagnostic and surveillance value in the identification of respiratory viruses supports the use of a combination of influenza testing and other RVP tests, or a multiplexed panel alone. The test volume, the proportion of specimens positive for influenza A and relative proportion of seasonal versus pH1N1 all affect the final cost, thus modeling for the optimal approach while fulfilling surveillance and diagnostic needs is complex. This movement to an RVP panel alone when influenza A prevalence is low would rely on effective near real-time surveillance systems that can provide decision makers the ability to analyze and review cleaned and interpreted laboratory data. In contrast, in a setting with a high prevalence of influenza, the laboratory leadership might decide, after consultation with the client base, whether only testing for influenza would be appropriate [22]. Another cost-saving approach is to stop H-typing of all specimens at the peak of the pandemic when essentially over 90% of the positive specimens were pH1N1 which needs to be balanced with ongoing monitoring and surveillance initiatives. During the peak week of the second pH1N1 wave, 19-40% of the total cost was used for H-typing of influenza A positive specimens (data not shown). Therefore, laboratory planning and preparedness should include policies and procedures that ensure smooth algorithm transitions at all pre-analytical, analytical and post-analytical steps of the testing process.

It should also be noted that DIAL has applications outside of the pandemic and can be used for health care and public health planning during routine respiratory seasons. The near-real time capability of this system provides up-to-date information of circulating respirator virus and is of great benefit for the trending of respiratory virus overtime.

Pandemic planning should be process focused with well established standard operating procedures to ensure that staff are able to handle transitions effectively without extensive micromanagement [23,24]. It is also important to have timely communications to the client base to indicate changes in algorithms during specific conditions and the impact of these changes on test ordering, clinical decision making and patient care [25].

Such real-time decision making requires an interactive and simple to use data management system that allows decision makers to have access to the most up-to-date laboratory data. A system such as DIAL is ideal in this setting as it harvests real time information from laboratory information systems and allows for analysis of aggregate data [26]. Access to this type of data may allow decision makers to potentially avoid decision making pitfalls such as; uncertainty, prejudice and optimism bias. However, the authors agree that biases will still exist even in the presence of DIAL, and decisions may still be made regardless of the data due to other factors impacting decision making such as group think, anchoring or choice-supportive bias [27].

## Conclusions

The authors believe that outright costs alone should not be the primary driving force in deciding which testing algorithm to choose. For example, an algorithm that utilizes the RVP alone is generally more cost effective than an algorithm combining both influenza testing and RVP. However, other factors such as test turn-around-time, burden on laboratory staffing hours, and overall algorithm performance characteristics should also be included in the decision making process. For example, a slightly more costly algorithm involving a combination of different tests may be chosen to ensure that tests results are returned to the clinician in a quicker manner as well as increased information and sensitivity. One may also argue that this increased laboratory test cost per specimen may be recovered in increased efficiency in other areas such as clinical decision making, infection control and public health practice. Furthermore, this "trade-off" must be clearly stated to those involved in global health budgets to avoid a narrow vision where laboratory costs are separated from other health costs.

A summary of the benefits and drawbacks of each scenario is included in Table 1. During peak periods when resources are limited and few mixed infections are seen (>60% influenza A), the authors believe that a scenario such as Scenario D (influenza A by CDC-M only with H-typing) focused on CDC-M will allow laboratories to provide the quickest results with less economic impact. This might be the last scenario carried out in a public health emergency when resources (both laboratory and human) become increasingly limited. In contrast, during periods of intermediate influenza A prevalence (40-60%), laboratories will still benefit from Scenario B (CDC-M first with H-typing & FluA-neg samples by RVP). Influenza A diagnosis turn-around-times can be decreased and most testing would not eliminate RVP testing. However, mixed influenza infections with other viruses would not be detected and the resources and space required to carry out this scenario may force some laboratories closer to Scenario D. In contrast, the use of RVP alone with H-typing (Scenario C) would be less expensive than other scenarios but would be very labor intensive and would increase turn-around times for influenza A diagnosis. One might utilize Scenario C when the prevalence of influenza A is <5% and the numbers of tests are limited. Such a protocol may be used after a pandemic period when the pandemic strain is known not to circulate, but care must be taken not used this methodology as an approach for surveillance of a new pandemic wave. There are new questions as to the value of using RVP alone to diagnose influenza A, especially when viral loads in patient specimens drop. Scenario A (RVP first with typing and influenza A negative samples by CDC-M and Typing) is the most expensive of all algorithms and is very labor intensive with high turn-around-times for influenza A diagnosis. However, this approach was critical and needed at the beginning of the pandemic when we were just learning about the performance characteristics of various diagnostic assays for the new influenza strain while obtaining data on total respiratory virus surveillance. Having access to near real-time cleaned and interpreted laboratory using DIAL allowed and enhanced ProvLab decision-making process so that a cost effective approach that provided good diagnostic and surveillance data (Scenario B) can be adopted early in the pandemic.

## List of Abbreviations

The following is a list of abbreviations; CDC-M: Centers for Diseases Control Influenza A Matrix Assay; CNPHI: Canadian Network for Public Health Intelligence; DFA: Direct fluorescent antibody; DIAL: data integration for Alberta laboratories; pH1N1: Pandemic (H1N1); ProvLab: Provincial Laboratory for Public Health; RT-PCR: Reverse transcriptase polymerase chain reaction; RVP: Luminex respiratory virus panel.

## Competing interests

The authors declare that they have no competing interests.

## Authors' contributions

All authors read and approved the final manuscript.

BL: undertook conceptualization, planning, data analysis, and writing manuscript. SM: conceptualization and critical revision of manuscript. JMH: data analysis and revising manuscript. SP: data analysis and revising manuscript. ML: conceptualization and revising manuscript. SD: conceptualization, planning, data analysis and writing manuscript.
